# Research Progress on Chronic Granulomatous Disease in Children

**DOI:** 10.7759/cureus.101159

**Published:** 2026-01-09

**Authors:** Zhixiu Zhang, Wanyi Li, Yongjun Wang

**Affiliations:** 1 The Second Department of Pediatric Respiratory, Gansu Provincial Maternity and Child-Care Hospital/Central Hospital in Gansu Province, Gansu Province, CHN

**Keywords:** cellular therapy, children, chronic granulomatous disease, immunodeficiency, laboratory diagnosis

## Abstract

Chronic granulomatous disease (CGD) is a rare primary immunodeficiency disorder characterized by defective neutrophil oxidative burst function, leading to impaired host defense against pathogenic microorganisms. This condition significantly increases the risk of invasive infections in affected patients. However, the clinical manifestations of CGD are not limited to infection-related complications; non-infectious complications can also lead to a variety of clinical symptoms. The global occurrence rate of CGD varies, and its inheritance patterns include X-linked and autosomal recessive forms. Research indicates that CGD patients are predominantly children who frequently face life-threatening infections and related complications, with early diagnosis often being challenging. Currently, hematopoietic stem cell transplantation (HSCT) is the only widely applied curative treatment for CGD. Recent advances in gene therapy offer a promising, safer, and more directly suitable alternative. This review primarily focuses on the pathophysiology, molecular genetics, occurrence rate, clinical manifestations, laboratory diagnosis, and cellular therapies of pediatric CGD, aiming to offer ideas for its clinical diagnosis.

## Introduction and background

Chronic granulomatous disease (CGD) was first reported in the 1950s in a 12-month-old child in Minnesota, who presented with a series of symptoms including chronic suppurative lymphadenitis, hepatosplenomegaly, pulmonary infiltrates, and eczematoid dermatitis, and was initially termed "fatal granulomatous disease of childhood" [1-3]. CGD is a rare primary immunodeficiency disorder characterized clinically by recurrent life-threatening bacterial and fungal infections and the formation of tissue granulomas. Its etiology involves defects in the genes encoding subunits of the nicotinamide adenine dinucleotide phosphate (NADPH) oxidase complex [2,4]. Under physiological conditions, the respiratory burst process is crucial for killing pathogens, and its dysfunction manifests as CGD [2]. Most patients with CGD are children, who frequently face life-threatening infections and inflammation-related complications [2]. In recent years, understanding of pathophysiology, diagnosis, and treatment of CGD has advanced significantly; however, achieving timely and accurate clinical diagnosis remains challenging. This review will focus on pathophysiology, molecular genetics, occurrence rate, clinical manifestations, and laboratory diagnosis, as well as cellular therapy and gene therapy for CGD.

## Review

Pathophysiology and molecular genetics

Chronic granulomatous disease (CGD) is a rare congenital immunodeficiency disorder caused by mutations in genes encoding components of the nicotinamide adenine dinucleotide phosphate (NADPH) oxidase complex [5]. The NADPH oxidase complex is essential for the generation of reactive oxygen species (ROS), which are critical for killing phagocytosed pathogens [5]. In CGD patients, impaired ROS production leads to two major consequences: first, the formation of characteristic systemic granulomas; second, a significantly increased susceptibility to bacterial and fungal infections, which can trigger uncontrolled inflammation and autoimmune responses [5]. Neutrophils from CGD patients are unable to effectively eliminate engulfed pathogens and fail to prevent the spread of infection [2]. Studies have shown that the NADPH oxidase complex is composed of two membrane proteins (gp91^phox^ and p22^phox^) and four cytosolic proteins (p47^phox^, p67^phox^, p40^phox^, and Rac1/2) [2,5-7]. It is noteworthy that the expression of the two membrane-bound components is interdependent; the absence of either one prevents the normal expression of the other [5].

Chronic granulomatous disease (CGD) is inherited via two distinct modes: X-linked recessive (XL) and autosomal recessive (AR) [6]. The pathogenesis of CGD involves five key genes: CYBB (encoding gp91^phox^, XL), NCF1 (encoding p47^phox^, AR), NCF2 (encoding p67^phox^, AR), NCF4 (encoding p40^phox^, AR), and CYBA (encoding p22^phox^, AR) [8]. Approximately 70% of cases are XL-CGD, which is caused by defects in the CYBB gene [8]. Mutations in the CYBB gene, located at Xp21.1, lead to X-linked CGD [9]. If a large deletion occurs in this region, patients may present with the McLeod phenotype, which can be accompanied by Duchenne muscular dystrophy or retinitis pigmentosa [9]. Among AR-CGD cases, mutations in the NCF1, NCF2, and CYBA genes are most common, with reported frequencies of approximately 20%, 5%, and 5%, respectively [10]. To date, only one patient with a p40^phox^ deficiency (NCF4 mutation) has been reported, presenting with a mild clinical phenotype limited to granulomatous colitis [10]. Furthermore, mutations in Ras-related C3 botulinum toxin substrate 2 (Rac2), a small GTPase involved in signal transduction from surface receptors to the NADPH oxidase complex, can cause CGD-like symptoms [10]. No cases of autosomal dominant CGD have been identified to date [10]. Notably, a study from the United Arab Emirates identified the same homozygous NCF1 variant, c.579G>A, in 12 out of 13 AR-CGD patients, suggesting it is a founder mutation for AR-CGD in that population [11]. On the other hand, mutations in the NCF2 gene, which encodes the cytosolic factor p67^phox^, also cause autosomal recessive CGD [12]. Roth et al. reported three patients carrying a novel NCF2 mutation, c.855G>C, who exhibited heterogeneous clinical phenotypes [12].

The stability of cytochrome assembly in the endoplasmic reticulum membrane is regulated by a chaperone known as Essential for Reactive Oxygen Species (EROS) [13]. EROS plays a critical role in immune surveillance by controlling reactive oxygen species (ROS)-mediated cellular killing through the modulation of the abundance of the ROS-generating enzyme NADPH oxidase 2 (NOX2) [13]. This protein is encoded by the CYBC1 gene, and its functional deficiency can also lead to chronic granulomatous disease (CGD) [13]. Additionally, the G6PD gene located on the X chromosome encodes glucose-6-phosphate dehydrogenase; inactivation of this enzyme results in insufficient NADPH production, thereby affecting NADPH oxidase activity [14]. Severe G6PD deficiency can also present with clinical manifestations similar to CGD [14]. In countries with low rates of consanguineous marriage, X-linked CGD (XL-CGD) is more common than autosomal recessive CGD (AR-CGD) [5]. In regions such as Egypt, the occurrence rate of autosomal recessive chronic granulomatous disease (AR-CGD) is generally high [15]. In clinical practice, priority should be given to ruling out CGD in any patient presenting with typical or atypical mycobacterial infection or bacilli Calmette-Guérin (BCG)-related disease [15]. Most patients with X-linked CGD develop symptoms before the age of 1 year, whereas AR-CGD patients often experience delayed onset, diagnosis, and intervention due to the preservation of partial oxidase activity [16]. When evaluating and treating CGD, full consideration should be given to the patient’s place of birth, climatic environment, and living conditions, as these factors may influence the spectrum of infectious pathogens and lead to infection types that deviate from conventional expectations [17].

Occurrence rate and clinical manifestations

Chronic granulomatous disease (CGD) is a rare primary immunodeficiency disorder, clinically characterized by recurrent severe bacterial and fungal infections, often accompanied by abscess formation, lymphadenitis, and granuloma formation. Most patients are diagnosed within the first 1 to 3 years of life [18]. The occurrence rate of CGD exhibits ethnic variations. Globally, it is estimated to be approximately 1 in 200,000 live births. However, among the Arab population in Israel, the occurrence rate is significantly higher, at approximately 1.5 per 100,000 live births [1,19,20]. Studies have shown that in the United States, approximately 20 new cases of CGD are diagnosed annually among 4 million newborns, while the number of new cases of severe combined immunodeficiency diagnosed each year is about 40 [3]. The estimated occurrence rates of CGD in Europe and Asia are similar [3]. The clinical manifestations of chronic granulomatous disease (CGD) are highly heterogeneous, with potential involvement of the skin, deep soft tissues, gastrointestinal tract, lungs, central nervous system, and virtually any other organ [21]. These often present as infectious or inflammatory complications, leading to the diagnosis of CGD across various medical specialties [21]. However, in clinical practice, the diagnosis of CGD is frequently challenging and prone to misdiagnosis. Contributing factors include atypical clinical presentations, rare pathogens, subtle manifestations of the genetic defect, rigid clinical thinking, and insufficient awareness of the disease [21]. The core features of CGD are severe recurrent bacterial and fungal infections and dysregulated inflammation [22]. Common bacterial pathogens include *Staphylococcus aureus* and the *Burkholderia cepacia* complex [22]. Among fungal infections, *Aspergillus* species are the most prevalent [19]. Patients with CGD are at extremely high risk for invasive *aspergillosis* (IA), with an occurrence rate of approximately 26%-45% [19]. Despite routine antifungal prophylaxis and targeted treatment, IA remains the most common infectious complication and the leading cause of death [19]. Inflammatory complications in CGD commonly manifest as inflammatory bowel disease and pulmonary lesions, with granuloma formation being a hallmark histological feature [23]. A medical center in Taiwan enrolled pediatric patients diagnosed with CGD between January 1999 and October 2021, including a total of nine children [24]. The median age at symptom onset and at diagnosis was found to be 0.92 years and 2.64 years, respectively [24]. Patients with X-linked (XL) CGD exhibited both earlier onset and earlier diagnosis compared to those with autosomal recessive (AR) CGD [24]. Skin and soft tissue abscesses were the most common sites of infection, with the primary pathogens being *Staphylococcus*, *Serratia*, and *Salmonella* species [24]. Furthermore, a case report documented that a CGD patient, following treatment for *Serratia*-induced osteomyelitis and lymphadenitis, developed subsequent bacteremia with a rare pathogen due to a significant reduction in neutrophil count [25]. A study from the United Arab Emirates involving 14 CGD patients reported a median age at symptom onset of 24 months but a median age at diagnosis of 72 months [11]. The most common clinical presentation in this study was lymphadenitis (71%), followed by abscess formation (57%), pneumonia (50%), invasive aspergillosis (21%), oral thrush (14%), and sepsis (14%) [11].

Most patients with chronic granulomatous disease (CGD) frequently present with infections, with common sites of infection including lymph nodes, lungs, liver, bones, and skin [26]. Clinically, it has been observed that the initial presentation in CGD patients is often lymphadenitis, while the occurrence rate of pneumonia in later stages can reach 70% to 80%, making it the most common type of infection [26]. Recurrent pulmonary infections from infancy can lead to damage of the lung parenchyma and bronchial structure, thereby affecting long-term pulmonary function [27]. Studies have found that the age at diagnosis for CGD patients typically ranges from 1.5 to 15 years, with pneumonia being the most common clinical manifestation, and some cases may present with bacilli Calmette-Guérin (BCG) disease [28]. In this cohort, four cases were autosomal recessive CGD (AR-CGD), three were X-linked CGD (X-CGD), and severe invasive infections caused by *Aspergillus*, *Staphylococcus*, and *Serratia* were observed, with the lungs and lymph nodes being commonly affected sites [28]. In addition to pneumonia, other pulmonary infections such as empyema have also been reported [26]. Abscesses are also a common initial presentation of CGD and can occur in any part of the body [26]. CGD should be considered in children with fever accompanied by abscesses, especially hepatic, perianal, or perirectal abscesses [26]. Among four genetically confirmed pediatric CGD cases summarized by Shandong Provincial Hospital, clinical manifestations included recurrent pulmonary infections, lymphadenitis, skin infections, and granuloma formation [29]. Pulmonary infections were relatively common, and most cases exhibited abnormal reactions following BCG vaccination [29]. Pulmonary imaging mainly showed multiple nodular high-density shadows, masses, or lobar consolidation with nodules, but cellular immunity and immunoglobulin levels were typically normal [29]. Radiologically, if multifocal/multilobar consolidation, mass-like consolidation, cavitary nodules, or necrotic lymphadenopathy are present, radiologists should be alert to the possibility of concomitant fungal infection [30]. Based on CYBB mutation types, X-linked CGD can be classified into two subtypes: X91^−^ and X91^+^, with the latter referred to as variant X-linked CGD [31]. Sun et al.'s analysis of Chinese patients with X91^+^ CGD showed that *Mycobacterium tuberculosis* infection is relatively common in this group, with approximately 50% of patients potentially developing BCG-related infections [31]. Overall, the incidence of tuberculosis and BCG-related complications is significantly higher in CGD patients than in the general population [32]. Therefore, screening for CGD should be performed in any patient presenting with adverse reactions to BCG vaccination, calcified left axillary lymph nodes, or persistent/recurrent/disseminated tuberculosis [32]. Furthermore, cutaneous manifestations are also important clues for CGD [33]. Anubha et al. found in their study that nasal red rash and papulopustular lesions (nasal sign) are relatively common in CGD patients and can serve as clinical features suggesting the diagnosis in patients with recurrent infections [33].

In addition to a markedly increased susceptibility to infections, patients with chronic granulomatous disease (CGD) frequently exhibit dysregulated inflammatory responses, characterized by excessive inflammation, which clinically necessitates the combined use of corticosteroids and anti‑infective agents [34]. Furthermore, CGD patients are prone to other autoinflammatory conditions, with colitis or inflammatory bowel disease (IBD) being particularly common [34]. It is estimated that nearly 50% of CGD patients develop concomitant IBD [35]. CGD‑associated IBD typically manifests during childhood, can involve any segment of the gastrointestinal tract, and shares pathological features with Crohn's disease. Rectal involvement is most frequently observed, and perianal or intra‑abdominal abscesses, as well as fistula formation, may lead to severe complications [36,37]. Thomas et al. reported a case of an 11‑year‑old child with CGD presenting with cutaneous and otorhinolaryngological symptoms, alongside biological and histological features consistent with Crohn's disease [38]. Further studies have revealed that CGD patients possess distinct gut microbiome and metabolome profiles [35]. Compared to healthy individuals, fecal samples from CGD patients show relative enrichment of bacterial taxa such as *Clostridium erysipelatos*, *Salmonella*, and *Clostridium guttiforme* [35]. Moreover, the microbiome and metabolome profiles of CGD patients with concomitant IBD (CGD‑IBD) differ from those of CGD patients without IBD, suggesting that gut microbial dysbiosis may be implicated in the pathophysiology of CGD‑IBD [35].

In recent years, some scholars have reported the occurrence of hemophagocytic lymphohistiocytosis (HLH) as a complication of chronic granulomatous disease (CGD), and it may even present as the initial clinical manifestation [39]. HLH is a life-threatening syndrome characterized primarily by multi-organ dysfunction, rapidly progressive cytopenia, hyperferritinemia, hypertriglyceridemia, and hypofibrinogenemia. Its pathological mechanism involves immune dysregulation and a cytokine storm [39]. Therefore, for pediatric CGD patients presenting with fever, timely recognition of HLH is crucial for subsequent management [40]. In summary, when CGD patients exhibit multi-system dysfunction and progressive cytopenia, HLH should be considered in the differential diagnosis [39]. Furthermore, case reports have indicated that CGD can be associated with glomerulonephritis. For instance, one case involved an adolescent male with newly diagnosed glomerulonephritis and a concomitant *Staphylococcus aureus* liver abscess, who was ultimately diagnosed with CGD [41].

Laboratory diagnosis of chronic granulomatous disease

The activity of phagocyte NADPH oxidase can be determined by activating purified peripheral blood neutrophils [42]. Activation methods include the use of serum-coated *Escherichia coli*, the addition of soluble stimulants such as phorbol myristate acetate (PMA) and formylmethionyl-leucyl-phenylalanine (fMLP/fMLF), and sometimes in combination with platelet-activating factor (PAF) [42]. Common detection methods include superoxide determination, hydrogen peroxide determination, and oxygen consumption measurement [42]. For the clinical diagnosis of chronic granulomatous disease (CGD), the NADPH oxidase activity of patient neutrophils is first assessed, followed by determination of the expression levels of each protein component of phagocyte NADPH oxidase. Additionally, the killing capacity of neutrophils against specific bacteria, yeasts, or fungi is evaluated [42]. Neutrophils isolated from CGD patients exhibit a functional defect in NADPH oxidase, resulting in an inability to produce reactive oxygen species (ROS) and consequently a loss of oxygen-dependent microbicidal activity [43]. Currently, the most widely used diagnostic techniques include dihydrorhodamine (DHR) flow cytometry analysis, the nitroblue tetrazolium (NBT) reduction test, and quantitative assays such as cytochrome c reduction and luminol-enhanced chemiluminescence [43,44].

The dihydrorhodamine (DHR) assay is a flow cytometry-based diagnostic method used to evaluate the activity of neutrophil NADPH oxidase [45]. The principle is that when neutrophils produce reactive oxygen species (primarily hydrogen peroxide) in response to stimulation by phorbol 12-myristate 13-acetate (PMA), the non-fluorescent dihydrorhodamine 123 is oxidized to fluorescent rhodamine 123 [45]. The latter can be quantitatively detected by flow cytometry, and the fluorescence intensity directly reflects the intracellular oxidative burst capacity [45].

The nitroblue tetrazolium (NBT) test is the most traditional and established method for diagnosing chronic granulomatous disease (CGD), achieved through a qualitative assessment of phagocyte NADPH oxidase activity [2]. The principle is based on the ability of normal phagocytes, upon ex vivo stimulation, to generate reactive oxygen species (primarily superoxide), which can reduce the yellow NBT to blue or black crystalline deposits within the cells [2]. The nitroblue tetrazolium (NBT) test is routinely performed on microscope slides, with manual assessment of the ratio of reduced (blue-black) cells to non-reduced (unstained) cells [2]. Under normal conditions, ≥95% of cells are positive. In patients with chronic granulomatous disease (CGD), the proportion of positive cells is significantly reduced or even absent due to defective superoxide production. The NBT test has certain limitations, including a relatively high risk of false-negative results, and its interpretation is susceptible to operator subjectivity [2]. To improve accuracy, each test must be performed concurrently with a healthy control sample to exclude errors caused by factors such as specimen handling [2]. Furthermore, the NBT test can be used to identify female carriers of X-linked CGD. Due to random X-chromosome inactivation (lyonization) in female carriers, both oxidase-normal and oxidase-deficient neutrophils can be detected in peripheral blood, resulting in a mixed population of positive and negative cells [26].

The quantification of superoxide anion (O₂˙⁻) generation was achieved through spectrophotometric determination of reduced cytochrome c [43]. The principle is based on the specific reduction of oxidized cytochrome c (ferric form) to reduced cytochrome c by O₂˙⁻, resulting in an increase in absorbance at 549.5 nm. The change in absorbance is proportional to the amount of O₂˙⁻ generated [43]. Superoxide dismutase (SOD) was added to a parallel system as a negative control to verify the specificity of the reaction; SOD inhibits the reduction of cytochrome c by scavenging O₂˙⁻ [43]. Using a millimolar extinction coefficient of 0.0211 μM⁻¹·cm⁻¹, the amount of extracellular O₂˙⁻ generated was calculated from the absorbance change [43]. In the experiment, SOD control tubes were included to correct for non-specific reduction of cytochrome c [43].

Luminol-enhanced chemiluminescence, as one of the common methods for clinical detection of reactive oxygen species (ROS) generation, offers advantages such as simple and rapid operation, high sensitivity, and low cell number requirement, enabling parallel detection of multiple samples under different stimulation conditions on the same microplate [43]. During detection, luminescence intensity is recorded every 1 to 5 minutes, with continuous monitoring for 2 hours, and the results are expressed in relative light units (RLU) [43]. Different stimulants induce characteristic luminescence kinetic curves [43]. By simultaneously testing normal controls and patient samples, the ROS generation capacity can be dynamically compared, and quantitative analysis can be performed by calculating the area under the curve (AUC) [43].

Through neutrophil function testing, abnormal results indicative of chronic granulomatous disease (CGD) are obtained, necessitating further genetic testing to confirm the diagnosis [2]. In clinical practice, sequencing of the patient's phagocyte oxidase (phox)-related genes is often performed to identify the specific molecular defect. The most common p47^phox^ subunit deficiency in CGD is frequently caused by homologous recombination between the NCF1 gene and its pseudogene, which may be missed by conventional standard sequencing methods; thus, confirmation via immunoblotting or gene dosage analysis is required [2]. Pathogenic variants in the CYBB gene encoding gp91^phox^ are diverse, including missense, nonsense, and promoter region mutations, as well as insertions, deletions, or splice site mutations. Among these, nonsense variants are associated with more severe clinical phenotypes and poorer prognosis [2]. Genetic testing not only confirms the diagnosis but also aids in prognosis assessment, and prediction of mortality risk, and provides clinical guidance for subsequent treatment options, such as hematopoietic stem cell transplantation or gene therapy [2]. Furthermore, more precise detection methods have been developed for common mutation types. Kuhns et al. established a droplet digital PCR (ddPCR)-based method utilizing two specific probes to distinguish between the wild-type (GTGT) and pathogenic ΔGT deletion sequences of the NCF1 gene. This ddPCR technique can effectively identify patients with p47^phox^-deficient CGD and their carriers [46]. Additionally, flow cytometry for detecting p47^phox^ protein expression can rapidly identify such patients [46].

Cellular and gene therapy for chronic granulomatous disease

Hematopoietic stem cell transplantation (HSCT) was first applied for the treatment of chronic granulomatous disease (CGD) in the early 1970s. However, HSCT was in an exploratory stage at that time and was rarely performed [23]. CGD itself is a life-threatening primary immunodeficiency disease that significantly reduces life expectancy, yet the decision to perform transplantation in CGD patients still faces numerous challenges [47]. Cases of rejection and graft failure observed in early transplantation experience indicated the necessity of using adequate conditioning chemotherapy and immunosuppressive regimens to reduce the risk of rejection [47]. Currently, HSCT has become the primary curative treatment for CGD, providing patients with donor-derived neutrophils possessing normal NADPH oxidase function and the capacity to generate superoxide anions [48-50]. Post-transplantation, not only is the patient's disease cured, but quality of life is also improved, with a survival rate reaching approximately 85%. Furthermore, most patients can discontinue long-term medication, achieving a state of complete cure [48]. Successful allogeneic HSCT can effectively eliminate chronic infections and inflammation while enhancing the patient's long-term quality of life [47]. CGD patients under eight years of age exhibit excellent clinical status following HSCT. However, younger patients require sufficient conditioning beforehand to prevent transplant rejection. For adolescent and adult patients, the risks and benefits of transplantation still require careful clinical evaluation [51].

In recent years, gene therapy has gradually gained attention as another potential curative strategy. In early clinical trials, approaches using γ-retroviral vectors yielded unsatisfactory results due to issues such as genotoxicity and difficulty in maintaining efficacy. However, gene therapy based on lentiviral vectors has shown promising initial outcomes [48]. A study conducted by Kohn et al. enrolled nine patients with X-linked CGD. Following myeloablative conditioning, these patients were infused with autologous CD34⁺ hematopoietic stem and progenitor cells transduced with a lentiviral vector carrying a functional CYBB gene [52]. The results indicated that all surviving patients did not develop new CGD-related infections, and six patients discontinued CGD-related antibiotic prophylaxis [52]. At the one-year follow-up, six of the nine patients achieved the primary study endpoint, demonstrating the effective therapeutic potential of lentiviral vector-based autologous gene therapy for patients with CGD [52]. Furthermore, advances in gene editing technology have provided new approaches for the treatment of CGD [53]. The conventional CRISPR/Cas system is limited in correcting certain CGD gene mutations due to its dependence on specific protospacer adjacent motif (PAM) sequences [53]. Vera et al. reported a PAM-independent base editing strategy capable of precisely targeting gene mutations in X-linked CGD [53]. This base editing approach supports progression toward first-in-human clinical trials and offers a potential therapeutic direction for treating other related immunodeficiency mutations [53].

## Conclusions

Although the genetic and phenotypic characteristics of chronic granulomatous disease (CGD) are well-defined and the molecular defects underlying CGD are clear, the consequences of reduced NADPH oxidase activity on immune dysregulation and inflammation are profound. These can lead to life-threatening infections and life-altering autoinflammatory symptoms, even in some asymptomatic carriers. Timely diagnosis, effective management, and early initiation of prophylactic treatment are essential. The emergence of new tools, such as genome editing technologies, will advance the understanding of CGD pathophysiology, facilitate exploration of other clinical manifestations of NADPH oxidase deficiency, and aid in the development of novel therapeutic targets. Gene therapy may represent a promising future approach for treating CGD. Further research is warranted regarding the pathophysiological mechanisms and the management of immunodeficiency in this condition.
